# Plasma REST: a novel candidate biomarker of Alzheimer’s disease is modified by psychological intervention in an at-risk population

**DOI:** 10.1038/tp.2017.113

**Published:** 2017-06-06

**Authors:** N J Ashton, A Hye, C A Leckey, A R Jones, A Gardner, C Elliott, J L Wetherell, E J Lenze, R Killick, N L Marchant

**Affiliations:** 1 Old Age Psychiatry, Institute of Psychiatry, Psychology and Neuroscience, Maurice Wohl Institute Clinical Neuroscience Institute, King’s College London, London, UK; 2 NIHR Biomedical Research Centre for Mental Health and Biomedical Research Unit for Dementia at South London and Maudsley NHS Foundation, London, UK; 3 Division of Psychiatry, University College London, London, UK; 4 VA San Diego Healthcare System, University of California, San Diego, San Diego, CA, USA; 5 Department of Psychiatry, University of California, San Diego, San Diego, CA, USA; 6 Department of Psychiatry, Washington University School of Medicine, St Louis, MO, USA

## Abstract

The repressor element 1-silencing transcription (REST) factor is a key regulator of the aging brain’s stress response. It is reduced in conditions of stress and Alzheimer’s disease (AD), which suggests that increasing REST may be neuroprotective. REST can be measured peripherally in blood plasma. Our study aimed to (1) examine plasma REST levels in relation to clinical and biological markers of neurodegeneration and (2) alter plasma REST levels through a stress-reduction intervention—mindfulness training. In study 1, REST levels were compared across the following four well-characterized groups: healthy elderly (*n*=65), mild cognitive impairment who remained stable (stable MCI, *n*=36), MCI who later converted to dementia (converter MCI, *n*=29) and AD (*n*=65) from the AddNeuroMed cohort. REST levels declined with increasing severity of risk and impairment (healthy elderly>stable MCI>converter MCI>AD, *F*=6.35, *P*<0.001). REST levels were also positively associated with magnetic resonance imaging-based hippocampal and entorhinal atrophy and other putative blood-based biomarkers of AD (*P*s<0.05). In study 2, REST was measured in 81 older adults with psychiatric risk factors for AD before and after a mindfulness-based stress reduction intervention or an education-based placebo intervention. Mindfulness-based training caused an increase in REST compared with the placebo intervention (*F*=8.57, *P*=0.006), and increased REST was associated with a reduction in psychiatric symptoms associated with stress and AD risk (*Ps*<0.02). Our data confirm plasma REST associations with clinical severity and neurodegeneration, and originally, that REST is modifiable by a psychological intervention with clinical benefit.

## Introduction

The repressor element 1-silencing transcription (REST) factor, alternatively named neuron-restrictive silencing factor (NRSF), is a protein that modulates neuronal differentiation and gene expression, and has recently been found to play an important role in Alzheimer’s disease (AD) neuropathology. Though highly expressed during development, REST was thought to be quiescent in the adult brain. However, recent observations indicate REST expression reactivated in the aging brain.^1^ REST has been shown to protect mature hippocampal neurons from toxic insults, for example, hyperexcitation,^2^ and to play a key role in regulating the aging brain’s response to stress.^1^ Furthermore, preclinical and clinical evidence demonstrate that reduced brain REST levels are associated with reductions in hippocampal volume and increased cognitive impairment.^1^ Conversely, individuals carrying significant amyloid- and tau-based neuropathologies, the classic defining hallmarks of AD, but who also have elevated REST, do not advance to a clinical diagnosis of dementia.^1^ These observations indicate that REST may act as a neuronal protective factor in older adults, by suppressing genes that drive neurotoxic processes resulting in cognitive decline and neuronal death.

REST presents a promising novel candidate biomarker for AD. However, its detection only in the central nervous system or in *in vitro* models has limited its utility as a translational research tool. Recently, however, REST has been identified in neuronally derived exosomes in blood, with diminishing levels shown to be clinically significant,^3, 4^ but the methods of exosome isolation can be a laborious and variable process thereby limiting its utility for translational research. These earlier studies have aimed to measure REST levels in neuronally derived exosomes by isolating them from plasma by affinity purification with an L1-CAM (CD171) antibody. While this protocol has been used routinely to successfully isolate exosomes, in this context it may prove problematic, given L1-CAM gene expression and splicing is known to be regulated by REST.^5^ Therefore, it is unclear how perturbed REST expression may alter the levels and forms of L1-CAM expressed on neuronal exosomes. To circumvent this, we elected to measure total peripheral REST in both membrane-bound vesicles and free forms simultaneously in patient plasma, particularly as the latter has yet to be fully explored. Our strategy therefore provides a highly comprehensive, reproducible and unbiased approach, which can be readily applied to large cohort-based studies.

The concept of cognitive debt identifies psychological risk factors for AD, including depression and anxiety, which are associated with dysregulated stress responses.^6, 7^ However, these risks have not been consistently associated with classic AD pathology.^6^ Cognitive debt supports a model of AD in which psychological stress is a contributory factor for the disease and proposes that reducing stress in individuals with high cognitive debt (for example, clinical depression and/or anxiety) will lower the incidence of AD. The identification of novel biomarkers that can provide a mechanistic/molecular link between these psychological risks and AD are needed to better understand the disease. REST is dysregulated in depression, a psychological ‘stress’ disorder with increased risk for AD.^8, 9^ Given its involvement in stress responses, REST may therefore be the biological mediator between psychological risk factors and AD.

Stress-reducing interventions may alleviate some of these dysregulated stress responses. Mindfulness-based stress reduction (MBSR) is an intervention that emphasizes focused, nonjudgmental awareness of present moment experiences.^10^ Preliminary findings in the elderly show that mindfulness-based training improves cognitive function in domains most sensitive to aging and AD,^11, 12^ and can reduce stress, anxiety and depression.^6, 13, 14^ Given that MBSR focuses on reducing stress, we hypothesized that this intervention would promote neural protection by targeting REST.

In this present study, we sought to detect circulating peripheral REST in two studies of older adults in order to investigate the following three questions: (1) are REST levels different between healthy elderly controls (HECs) and patients with mild cognitive impairment (MCI) and AD; (2) and are they associated with surrogate markers of AD; (3) can REST levels be modified by a stress-reducing intervention in older adults with high cognitive debt.

## Materials and methods

### Cohorts

#### AddNeuroMed

The AddNeuroMed (ANM) project, part of InnoMed (Innovative Medicines in Europe), is a multicenter European program designed to develop and validate novel surrogate markers of AD that combines magnetic resonance imaging (MRI) data with other putative biomarker and clinical data. Detailed information on the study design and enrollment procedures of ANM have been previously discussed.^15^ The diagnosis of probable AD was made as follows. Inclusion criteria: (1) ADRDA/NINCDS and DSM-IV criteria for probable AD. (2) Mini Mental State Examination (MMSE) scores ranging between 12 and 28. (3) Age 65 years or above. Exclusion criteria: (1) significant neurological or psychiatric illness other than AD. (2) Significant unstable systemic illness or organ failure. All AD participants had a Clinical Dementia Rating scale score of 0.5 or above. The diagnosis of MCI was made according to the Peterson criteria.^16^

#### Intervention cohort

Participants were 103 adults aged 65 or older, 81 of whom provided blood samples before and after the intervention. Details of this cohort and the intervention have previously been reported.^17^ Briefly, participants with (1) clinically significant anxiety or depressive symptoms, and (2) a current diagnosis of a depressive and/or anxiety disorder and (3) who endorsed current subjective aging-related neurocognitive problems were recruited into a clinical trial to examine effects of MBSR versus a comparison condition on cognitive function and psychiatric symptoms. Exclusion criteria: a diagnosis of dementia or dementia symptoms (ascertained from the Short Blessed Test^18^), prescription of cognitive enhancing medication, alcohol or substance use disorders within the past 6 months, current or lifetime psychotic or bipolar disorder, current participation in psychotherapy or regular engagement in mindfulness practice or yoga, corticosteroid use, and serious medical illness that would prevent study participation or accurate data collection (for example, congestive heart failure, oxygen dependent). Individuals currently taking antidepressants or anxiolytics were eligible if they had been on a stable dose for at least a month prior to enrollment and agreed to remain stable throughout the intervention.

### Interventions

Participants were randomized in groups of five to eight people to either MBSR or to a health education control condition. The 8-session MBSR intervention was conducted according to the protocol developed by Jon Kabat-Zinn,^19^ modified for older patients, along with a half-day meditation retreat. The health education comparison intervention was based on the health care self-management book written by Lorig *et al.*^20^ The 8-session program included topics on understanding and managing common conditions and symptoms, healthy eating, managing medications and communicating with health care providers. Both the MBSR and health education programs were once weekly, group-based sessions of ~90 min. Participants in both groups received manuals and had between-session assignments (more details about the interventions can be found in Lenze *et al.*^17^).

### Blood collection and processing

Blood samples from both cohorts were drawn by venipuncture at the time of assessment (baseline and post intervention for the Intervention cohort). ANM participants were required to fast for at least 2 h before collection. Blood samples were centrifuged at 3000 *g* for 10 min at 4 °C. Blood samples from the Intervention cohort (non-fasting) were centrifuged at 3000 *g* for 15 min. Plasma supernatant was collected, divided into aliquots and frozen at −80 °C until further use. All samples were centrifuged within 2 h of collection. The APOE single-nucleotide polymorphisms (SNPs) rs429358 and rs7412 were genotyped using Taqman SNP genotyping assays (determined by allelic discrimination assays based on fluorogenic 5′ nuclease activity) and the allele inferred.

### Immunological assays

Plasma REST was quantified using a sandwich enzyme-linked immunosorbent assay, Cusabio, American Research Products (College Park, MD, USA), in both studies. Plasma samples were pretreated with Tween-20 (4% v/v) to release REST contained within extracellular vesicles and mixed on an orbital shaker for 15 min prior to primary antibody incubation. Plasma samples were measured in duplicate and mean absorbance values (450 nm) exported into Sigma plot (Systat Software, London, UK; version 12) for estimation of protein concentrations using a five-parameter logistic fit. To assess the performance of the enzyme-linked immunosorbent assay the intra- and inter-assay coefficient of variance (CV%) were calculated for both the cohorts. The investigator who ran the immunological assays was blind to clinical condition (in the ANM cohort) and intervention group (in the Intervention cohort).

Other candidate plasma proteins had been previously assayed in the ANM cohort by using multiplex bead assays (Luminex xMAP, Austin, TX, USA).^21^ We investigated the association of REST with 16 proteins observed as markers of AD disease severity and conversion^21^ in the ANM cohort. Proteins associated with REST in the ANM cohort were then examined in the Intervention cohort using the same technique.

### Cognitive and clinical assessments

In the Intervention cohort, memory was assessed by immediate and delayed paragraph and list recall.^22^ Executive function was assessed by the Delis–Kaplan Executive Function System (DKEFS) Verbal Fluency test and the DKEFS Stroop test.^23^ These cognitive assessments were conducted at baseline and post intervention (8 weeks). For each time point, composite scores for memory and executive function were created by averaging *Z*-scores for each measure. The Wechsler Test of Adult Reading (WTAR)^24^ was administered to assess premorbid neurocognitive function at baseline. Clinical assessments made using subscales of the PROMIS questionnaire included assessments of subjective cognitive concerns, anxiety and depression^25^ Chronic worry was assessed by the Penn State Worry Questionnaire-Abbreviated.^26^ Clinical assessments conducted at baseline and post intervention (8 weeks) were used for analyses.

### Magnetic resonance imaging

A description of MRI procedures in the ANM cohort has been previously described.^21^ High-resolution sagittal 3D T1-weighted magnetization prepared rapid gradient-echo (MPRAGE) volume (voxel size 1.1 × 1.1 × 1.2 mm^3^) was acquired on 1.5 T MRI scanners for 148/195 of the ANM sub-cohort.^27^ Measures of hippocampal volume, entorhinal cortex volume, ventricular volume and whole brain volume were chosen as MRI endophenotypes of AD. Participants in the Intervention cohort did not receive MRI scans.

### Statistical analysis

Statistical analyses and figures were processed using SPSS 22 (IBM, Armonk, NY, USA) and R software. REST concentrations in both cohorts were non-normally distributed and were log_10_-transformed except for ANM REST in the Tobit model. When reporting REST mean and s.d.’s, we report the original pg ml^−1^ values.

Age, sex, assay plate, plasma storage duration (days) and center were examined as covariates in the ANM cohort. REST was significantly affected by assay plate and center. To analyze differences in REST in the ANM cohort, we implemented a Tobit model, using a left-censored value of 25.63 (the lowest recorded REST pg ml^−1^), diagnosis (controls, cMCI, sMCI and AD) as a predictor variable, and adjusted for dummy variables assay plate and center code. The relationship between APOE and REST was examined separately using non-parametric testing.

In the Intervention cohort, REST was significantly affected by assay plate and center, and age differed between the two conditions; therefore REST values were adjusted for these variables using a generalized linear regression model (GLM). All subsequent analyses were performed on the GLM-adjusted (non-standardized residuals) data. To analyze differences in REST pre- and post intervention in the Intervention cohort, we used a linear-mixed model (fitted with restricted maximum likelihoods) with intervention condition (MBSR and health education) and time (pre- and post intervention) as predictor variables, condition × time as an interaction term and time grouped by participant as random effects. For this analysis, we are primarily interested in whether the pre- and post-intervention changes in REST concentration change depending on condition. *Post hoc* analyses of interactions for the Intervention cohort were performed using pairwise comparison of least square means of log-transformed REST values; non-adjusted *P*-values are reported. REST, cognitive and clinical data in the Intervention cohort were also divided into ‘change’ categorical variables (increase, decrease) by subtracting baseline from post-intervention levels. If a REST was below the detectable range at baseline and was within the detectable range post intervention this was classified as an ‘increase’, likewise a sample was classified as ‘decrease’ if it was detectable at baseline but not post intervention. Chi-square analyses were conducted on categorical data.

Partial correlation analysis was performed to examine REST level associations with structural MRI brain imaging (ANM only), baseline cognitive and clinical data (Intervention cohort) or additional proteomic data (ANM and Intervention cohort). Benjamini–Hochberg *Q*-values were calculated as a multiple testing correction.

## Results

### REST and clinical group (AddNeuroMed)

The impetus for the present study was to investigate REST as a possible blood-based biomarker of the stress response mechanism in AD. To achieve this aim, we examined whether REST was detectable in blood (plasma), and if patients classified as HECs and clinically defined AD showed differential REST expression. In a feasibility study (*n*=62; Supplementary Table S1) in the ANM cohort, we demonstrated that REST levels were quantifiable in plasma in 71% of the sample. Further, REST levels were reduced in AD (*M*=46.77 pg ml^−1^, s.d.=2.05) compared to HEC (*M*=89.13 pg ml^−1^, s.d.=2.63; *t*(60)=3.03, *P*=0.003) and significantly more AD REST levels were below the limits of detection than HEC (*χ*^2^(1)=7.82, *P*=0.005). Due to a number of the AD samples falling below the detection level, we further investigated the prospect of REST being located in extracellular vesicles—this has been previously reported.^3, 4^ By pre-treating plasma samples, we sought to mobilize REST from microvesicles, thereby releasing REST into the soluble space. In the AD group a 61.39% increase in REST signal (*M*=110.01 pg ml^−1^, s.d.=2.63) and increase in the number of samples above limits of detection were observed. The HECs group also demonstrated a substantial increase in REST signal (115.17%, *M*=222.43 pg ml^−1^), thus demonstrating REST as both contained within microvesicles and circulating freely in blood (plasma). This is of particular significance, as previous studies have focused mainly on REST contained in microvesicles and therefore have not measured free REST levels.

We then sought to confirm this initial finding in a significantly larger selection of participants from the ANM cohort (*n*=195), expanded to include individuals with MCI who remained stable (sMCI) or later converted to AD (cMCI). Table 1a describes the demographic characteristics of ANM. An even distribution of clinical diagnosis was selected (AD=65, sMCI=35, cMCI=30, HEC=65), matched for age and sex.

**Figure 1:**
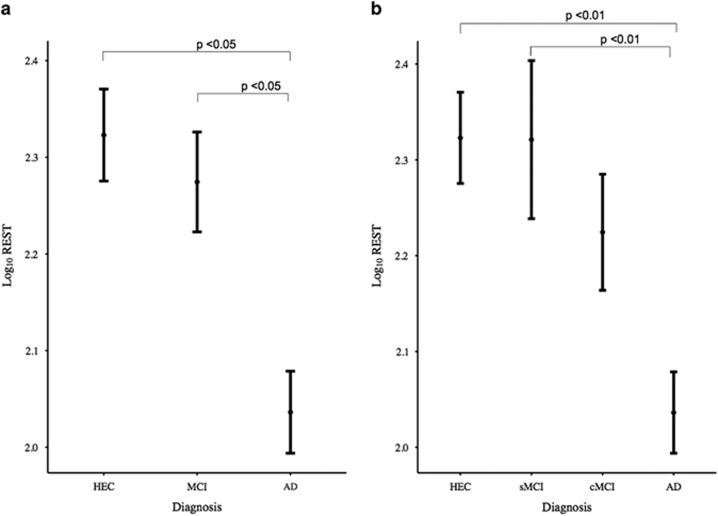
Repressor element 1-silencing transcription (REST) values stratified by clinical diagnosis. Log_10_-transformed REST values (corrected for assay plate and center) are represented as means (s.e.m. error bars). (**a**) There was a significant decline in REST from healthy elderly controls (HEC) to mild cognitive impairment (MCI) to Alzheimer’s disease (AD). Compared with the AD group, the MCI group showed significantly higher levels of REST (+95.73, 95% CI 1.82–189.65, *P*=0.046) as did the control group (+122.45, 95% CI 27.96–216.93, *P*=0.011). (**b**) Dividing mild cognitive impairment groups into those who remained stable (sMCI) and those who later converted to AD (cMCI), compared with the AD group, REST levels were not significantly different in the cMCI group (26.00, 95% CI −90.67–142.68, *P*=0.66) but were significantly higher in the sMCI group (159.06, 95% CI 47.77–270.65, *P*=0.005) and controls (126.12, 95% CI 32.53–219.70, *P*=0.008).

In total, 168/195 (86%) participants had quantifiable REST and intra- and inter-assay were 9.2% and 14.6% CV, respectively. The AD group as before had lower REST levels (*M*=112.12 pg ml^−1^, s.d.=2.21) than HECs (*M*=199.21 pg ml^−1^, s.d.=2.32). The MCI group as a whole showed lower REST compared with HECs group (*M*=193.51 pg ml^−1^, s.d.=2.56). There was a significant decline in REST from HECs to MCI to AD (Figure 1a). Using the AD cohort as the comparator, the MCI group showed significantly higher levels of REST (+95.73, 95% CI 1.82–189.65, *P*=0.046) as did the control group (+122.45, 95% CI 27.96–216.93, *P*=0.011).

We next divided the MCI group into those who remained stable (sMCI, *M*=207.83 pg ml^−1^, s.d.=3.03) and those who later converted to AD (cMCI, *M*=179.23 pg ml^−1^, s.d.=2.06) and repeated the analyses. Using the AD group as the comparator, REST levels were not significantly different from the cMCI group (26.00 pg ml^−1^, 95% CI −90.67 to 142.68, *P*=0.66); however, the sMCI group (159.06 pg ml^−1^, 95% CI 47.77 to 270.65, *P*=0.005) and controls (126.12 pg ml^−1^, 95% CI 32.53 to 219.70, *P*=0.008) had significantly higher levels of REST (Figure 1b). APOE genotype had a significant effect on REST levels (*U*=2733.00, *P*=0.02) when examined in isolation, but adding genotype as a covariate to the between group comparisons did not alter the findings.

### REST and surrogate AD markers

Partial correlation analyses, co-varying for APOE genotype, demonstrated associations with hippocampal volume, entorhinal cortex volume, whole brain volume and a trend toward significance for ventricular volume (Table 2). In addition, we investigated REST level associations with 25 plasma proteins previously associated with either cognitive decline or disease severity.^21^ REST had a positive correlation with ApoC3 and ApoA1, brain-derived neurotrophic factor (BDNF), regulated on activation, normal T cell expressed and secreted (RANTES), plasminogen activator inhibitor-1 (PAI-1) and neuron-specific enolase (NSE) (Table 2). After adjusting for multiple comparisons, REST associations with hippocampal volume, entorhinal cortex volume, whole brain volume, BDNF, RANTES, PAI-1 and NSE remained statistically significant (Table 2).

### REST modification (Intervention cohort)

Demographic, clinical and cognitive characteristics of participants randomized to the MBSR condition were not statistically different from those in the control condition; however, they were aged slightly younger (Table 1b).

REST was detectable in 52 baseline samples and 51 post-intervention samples, with 37 participants having quantifiable REST at both time points and performed in line with the ANM cohort with 8.5% (intra) and 12.3% (inter) CV. Correlation analyses revealed that baseline REST was not associated with anxiety or depressive symptoms, worry, cognitive concerns, scores on the short blessed cognitive test or measures of executive function or memory (*P*’s>0.05).

**Figure 2:**
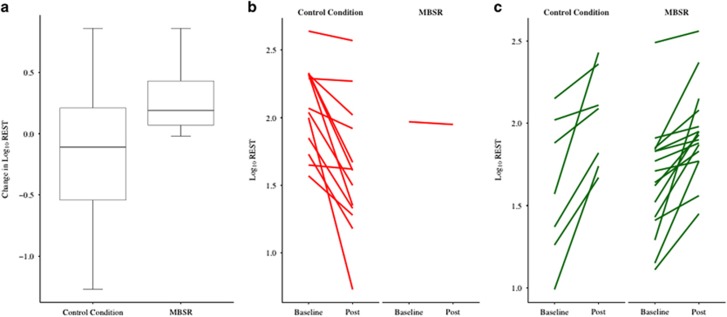
Change in repressor element 1-silencing transcription (REST) values stratified by intervention condition. Log_10_-transformed REST values were corrected for assay plate, center and age. There was a significant interaction between condition and time (*F*=5.78, *P*=0.02). (**a**) Box and whiskers plot of change in mean REST levels (post intervention minus baseline). (**b**) Spaghetti plot of baseline and post intervention REST levels of participants who showed a decrease in REST. (**c**) Spaghetti plot of baseline and post intervention REST levels of participants who showed an increase in REST.

We tested whether changes in REST occurred between pre- and post intervention (time) differently for the MBSR group compared to controls. We found a significant interaction between condition and time (*F*=5.78, *P*=0.02). Initially there was significant difference in REST between MBSR patients and controls pre-intervention (−0.23, s.e.=0.10, *P*=0.027, REST difference −34.10 pg ml^−1^) but not post intervention (−0.06, s.e.=0.10, *P*=0.504, REST difference −9.52 pg ml^−1^). This is due to a near-significant increase in REST pre-intervention to post intervention for MBSR patients (0.19, s.e.=0.10, *P*=0.055, REST difference +38.70 pg ml^−1^), which was not present for pre- to post changes in REST for the controls (−0.11, s.e.=0.08, *P*=0.178, REST difference −8.60 pg ml^−1^, Figure 2a). Individual data are shown in Figures 2b and c. Controlling for sex and/or APOE status did not alter the results.

**Figure 3:**
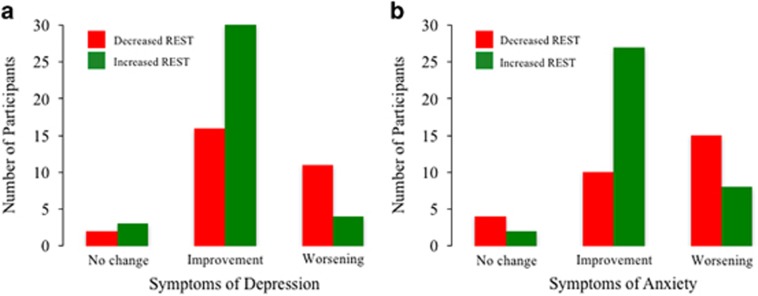
Frequency plot of participants showing an increase or decrease in repressor element 1-silencing transcription (REST) after the intervention, stratified by change in psychiatric symptoms of depression and anxiety. An increase in REST was significantly associated with improvement in symptoms of (**a**) depression (*χ*^2^=6.86, *P*=0.03) and (**b**) anxiety (*χ*^2^=9.78, *P*=0.008).

Treating data as categorical variables, an increase in REST was significantly associated with improvement in symptoms of depression (*χ*^2^=6.86, *P*=0.03, Figure 3a) and anxiety (*χ*^2^=9.78, *P*=0.008, Figure 3b), irrespective of intervention condition, but not with memory, executive function, cognitive concerns or worry.

In this data set, we independently confirmed the strong association of REST with other candidate plasma proteins of cognitive decline at baseline: BDNF (*r*=0.5, *P*<0.001), RANTES (*r*=0.421, *P*=0.002) and PAI-1 (*r*=0.321, *P*=0.026). However, none of these putative markers displayed any significant change after the intervention.

## Discussion

This study indicates that blood REST protein levels are a novel, psychologically modifiable candidate biomarker of cognitive decline and AD.^28^ Peripherally circulating REST levels declined with increasing clinical severity, resulting in AD patients showing the lowest levels. Patients with MCI who later converted to AD exhibited an ‘Alzheimer’s-like’ REST profile, whereas non-converters (stable) MCI participants had a REST profile that resembled HECs. Importantly, we demonstrated that REST levels can be modulated through a relatively brief behavioral stress-reduction intervention, and that changes in REST are associated with clinical improvements.

Our finding of reduced REST in AD participants mirrors the neural loss of REST in autopsy-confirmed AD patients.^1^ In support of these findings, a decrease in REST levels contained within neuronally derived exosomes has previously been reported in small sample of patients with AD and MCI compared with elderly participants.^3, 4^ Here we demonstrated that a substantial proportion of REST is located outside these extracellular vesicles and is free-floating in plasma. By releasing membrane-enclosed REST into the soluble space, we measured total REST and increased the signal of REST considerably, contributing to the strong linear decline of REST with increasing severity of cognitive deficits and clinical diagnosis. It must be acknowledged, however, that REST can be expressed in multiple cell types in the periphery, so until a direct comparison of peripheral and central REST levels can be made, it remains unclear whether REST measured here indeed reflect levels in the central nervous system. Nevertheless, these findings strongly support that our method is capable of measuring low abundant proteins quickly and efficiently, thus avoiding the time consuming preparation of exosomes. Our unbiased approach also circumvents technical limitations of exosome enrichment particularly when using neuronal adhesion molecules, such as L1CAM, whose expression and splicing are regulated by REST.

Using MRI as a surrogate marker of disease pathology, we found REST associations with several brain regions impacted early in AD—hippocampal and entorhinal cortices. Controlling for APOE genotype did not alter the association with REST and MRI measures or clinical status, indicating that REST may be a specific marker of AD risk, independent of APOE genotype.

Investigating the association of REST with previously identified plasma protein markers of MCI conversion to AD and cortical atrophy,^21^ we found strong associations with BDNF, RANTES, PAI-1 and NSE. These associations were confirmed, independently, in the Intervention cohort. There is evidence that, like REST, BDNF has a neuroprotective role in the presence of pathological conditions.^29^ Decreased BDNF expression in the AD brain has been shown^30^ and several studies have observed a decrease of peripheral BDNF in MCI and AD patients,^31, 32, 33^ including in a meta-analysis of 7277 subjects.^34^ Indeed, higher BDNF serum levels have shown to protect against the future incidence of AD.^35^ In addition, sequence variations in the BDNF gene have been confirmed to be connected with neurological disorders such as major depression.^36^ While recently, both REST and BDNF have been associated with cognitive dysfunction in epilepsy sufferers.^37^ In this study, we have shown a decrease in BDNF in AD and MCI, and a strong relationship between REST and BDNF in two independent cohorts. NSE in blood is considered to be a peripheral biomarker of neuronal injury^38^ and blood–brain barrier dysfunction,^39^ and PAI-1 has been strongly linked with aging,^40^ remitted depression^41^ and severe anxiety.^42^ Given the relationship we have shown between these three markers and REST plus their involvement in psychological disorders carrying increased risk for AD, in combination, REST, BDNF, NSE and PAI-1 may provide a more sensitive predictor of cognitive decline than a single peripheral protein.

Of major interest we report that in older adults at risk of developing dementia, a significant increase in REST levels was induced by a stress reduction intervention after just 8 weeks, potentially highlighting REST as a modifiable target. REST is known to highly regulate the cortisol levels via the modulation of CYP11B1 gene,^43^ thus, the intervention may have modified cortisol levels, which in turn influenced the REST levels. Furthermore, individuals showing an increase in REST also had a reduction in symptoms of anxiety and depression, which indicates a mechanistic link between REST, stress and psychological risk factors for AD. These findings are also supported by previous research showing a relationship between reduced REST expression and depression,^8^ and suggest that interventions targeting REST would have clinical utility. No association was found between REST and cognition, possibly as the intervention was too brief for such changes to occur or the limited ability to detect cognitive improvements.

A larger study with a longer follow-up will be needed to clarify the clinical implication of this REST increase with MBSR, but our study has demonstrated, using a rigorously controlled design, that REST does in fact increase with MBSR.

The participant-inclusion criteria of the two cohorts included in this study either exclusively recruited individuals without the presence of a psychiatric disorder (for example, depression, anxiety, ANM) or with a psychiatric disorder (Intervention cohort), therefore in this study we were unable to directly compare REST levels in depressed/anxious versus healthy older adults. Due to this constraint, the direct link between cognitive debt and this novel biological marker has not yet been investigated.

Population-based measures of REST in varying ages and clinical conditions need to be refined before any cutoff of clinical significance is proposed. The development of an assay that is robust and consistent in quantifying low values of REST would be a critical first step. Nonetheless the results are a promising indicator that reducing stress raises REST levels in blood and, by inference may also increase levels in brain too; something we aim to determine in future studies.

Notwithstanding the above limitations, we were able to show that REST is associated with disease state and surrogate markers for disease, REST can be modified using a behavioral intervention and that increased REST is associated with a reduction in psychological risk factors for AD, indicating the utility of this marker. Indeed, the failure of the brain’s stress response system via REST is now being considered as an alternative therapeutic target for AD.^1, 44^ A search for a blood-based surrogate of AD pathology, in particular neocortical amyloid burden, to compliment participant recruitment into AD therapeutic trials is ongoing.^45, 46^ Aging individuals who exhibit substantial AD pathology appear to be protected from dementia when neuronal REST levels are high and therefore, AD structural pathology may not be sufficient to cause dementia. The findings presented here are of great significance for the diagnosis and clinical management of AD, as determining REST in blood provides a prognostic marker that can help stratify patients for inclusion in trials, identify those at high risk of ‘stress-induced’ cognitive decline and determine the efficacy of therapeutic interventions. Dysregulation of REST provides an insight into the underlying processes leading to AD by which aging factors in the brain lead to cognitive decline. Therefore, further elucidation of these mechanisms will provide targets for the development of novel therapeutic strategies.

## Figures and Tables

**Table 1:** Demographic and clinical data from (a) ANM sub-cohort and (b) Intervention sub-cohort

*(a) ANM sub-cohort*	*HEC*	*sMCI*	*cMCI*	*AD*	P*-value*
Participants, *n* (%)	65 (33)	36 (18)	29 (15)	65 (33)	NS
Age, mean years (s.d., range)	75.7 (8.6, 53–89)	76.8 (8, 65–89)	75.4 (7.1, 57–89)	76.8 (8, 61–90)	NS
Sex, *n* females (%)	33 (50.8)	15 (41.7)	17 (58.6)	33 (50.8)	NS
MMSE, mean (s.d., range)	29 (1.1, 27–30)	27 (1.9, 21–30)	25.7 (2.4, 18–30)	19.6 (4.1, 12–26)	<0.001^a^
CDR sum of boxes, mean (s.d., range)	0.01 (0.1, 0–0.5)	0.47 (0.1, 0–0.5)	0.48 (0.1, 0–0.5)	1.3 (0.7, 0.5–3)	<0.001^b^
APOE ε4 carrier, *n* (%)	23 (35.4)	9 (25.7)	17 (56.7)	32 (49.2)	<0.05^c^
Years to conversion, mean (s.d., range)	NA	NA	1.06 (0.22, 0.95–2.11)	NA	NA

*(b) Intervention sub-cohort*	*Total sample*	*MBSR*	*Health education*	P*-value*
*Demographics*				
Participants, *n* (%)	81 (100)	30 (37)	51 (63)	
Age, mean (s.d., range)	72.1 (5.7, 65–89)	70.2 (4.2, 65–79)	73.3 (6.2, 65–89)	0.03
Sex, *n* females (%)	58 (71.6)	22 (73.3)	36 (70.6)	NS
Education, mean years (s.d., range)	15.8 (2.8, 6–20)	16.0 (3.0, 6–20)	15.7 (2.6, 8–20)	NS
APOE ε4 carrier, *n* (%)	26 (32.1)	10 (33.3)	16 (31.4)	NS
				
*Baseline clinical and cognitive measures*				
Short Blessed, mean (s.d., range)	1.7 (2.1, 0–9)	1.7 (1.6, 0–6)	1.9 (2.3, 0–9)	NS
WTAR Raw, mean (s.d., range)	40.2 (8.4, 8–50)	39.3 (8.5, 23–49)	40.6 (8.4, 8–50)	NS
PROMIS anxiety, mean (s.d., range)	20 (5.8, 7–31)	20.7 (5.8, 8–31)	19.6 (5.8, 7–29)	NS
PROMIS depression, mean (s.d., range)	19.9 (7.2, 8–36)	20.5 (7.9, 8–36)	19.6 (6.8, 8–35)	NS
PROMIS cognitive concerns, mean (s.d., range)	23.2 (7.4, 8–40)	24.2 (9.0, 8–40)	22.7 (6.2, 10–36)	0.004
Penn State Worry Questionnaire-Abbreviated, mean (s.d., range)	27.6 (7.7, 8–50)	28.4 (6.4, 9–37)	27.2 (8.3, 8–40)	0.09

Abbreviations: AD, Alzheimer’s disease; ANM, AddNeuroMed; CDR, Clinical Dementia Rating; cMCI, converting mild cognitive impairment; HEC, healthy elderly control; MBSR, mindfulness-based stress reduction; MMSE, Mini Mental State Examination; NA, not applicable; NS, not significant; sMCI, stable mild cognitive impairment; WTAR, Wechsler Test of Adult Reading.

a Significant across all four groups.

b Significant across all groups except between sMCI and cMCI.

c Significant only between sMCI and cMCI.

**Table 2:** MRI brain structures and candidate plasma proteins of cognitive decline and disease risk associated with REST values in the ANM cohort

	*df*	*Correlation coefficient with REST*	P*-value*	Q*-value*
*MRI brain structures*				
Mean hippocampal volume	122	0.246	0.013	0.025
Mean entorhinal cortex volume	122	0.295	0.003	0.013
Ventricle volume	122	−0.179	0.060	NS
Whole brain volume	102	0.213	0.030	0.038
				
*Significant candidate plasma proteins*^21^				
ApoA1	164	0.178	0.022	NS
ApoC3	162	0.162	0.039	NS
BDNF	125	0.267	0.002	0.006
NSE	162	0.284	0.001	0.004
PAI-1	159	0.286	>0.001	0.002
RANTES	162	0.224	0.004	0.008

Abbreviations: ANM, AddNeuroMed; ApoA1, Apolipoprotein A1; ApoC3, Apolipoprotein C3; BDNF, Brain-derived neurotrophic factor; MRI, magnetic resonance imaging; NS, not significant; NSE, neuron-specific enolase; PAI-1, plasminogen activator inhibitor type 1; RANTES, regulated on activation, normal T cell expressed and secreted; REST, repressor element 1-silencing transcription.
