# Supplementary material for: Plasma REST: a novel candidate biomarker of Alzheimer’s disease is modified by psychological intervention in an at-risk population
Source: Transl Psychiatry. 2017 Jun 6;7(6):e1148–. doi: 10.1038/tp.2017.113 (PMC5537638; doi:10.1038/tp.2017.113)
Supplement: Supplementary Table S1 [file tp2017113x1.docx]

Supplementary Data

Table S1: Demographic details of the feasibility study

|  | Alzheimer’s disease (AD) | Control  (HEC) | P-value |
| --- | --- | --- | --- |
| Number of participants (*n*) | 31 | 31 |  |
| Gender; females (*n* (%)) | 17 (55%) | 18 (58%) | 0.40 |
| Age in years (mean (SD)) | 77.85 (8.08) | 77.10 (7.26) | 0.40 |
| APOE ε4 carrier (*n* (%)) | 17 (55%) | 11 (36%) | 0.047 |
| MMSE (mean (SD)) | 20.7 (5.36) | 29 (0.8) | <0.001 |
